# A descriptive qualitative examination of knowledge translation practice among health researchers in Manitoba, Canada

**DOI:** 10.1186/s12913-017-2573-9

**Published:** 2017-09-06

**Authors:** Kathryn M. Sibley, Patricia L. Roche, Courtney P. Bell, Beverley Temple, Kristy D.M. Wittmeier

**Affiliations:** 1 Department of Community Health Sciences, Rady Faculty of Health Sciences, University of Manitoba, 379 – 753 McDermot Avenue, Winnipeg, MB R3E 0T6 Canada; 2 George and Fay Yee Centre for Healthcare Innovation, 379 – 753 McDermot Avenue, Winnipeg, MB R3E 0T6 Canada; 3 College of Nursing, Rady Faculty of Health Sciences, University of Manitoba, 483 Helen Glass Centre for Nursing, 89 Curry Place, Winnipeg, MB R3T 2N2 Canada; 4 Department of Physiotherapy, Winnipeg Health Sciences Centre, 183A-800 Sherbrook Street, Winnipeg, MB R3A 1S1 Canada; 5 Department of Pediatrics and Child Health, Rady Faculty of Health Sciences, University of Manitoba, 183A-800 Sherbrook Street, Winnipeg, MB R3A 1S1 Canada

**Keywords:** Knowledge mobilization, Exchange, Transfer, Implementation science, Semi-structured interviews, Qualitative analysis, Health research

## Abstract

**Background:**

The importance of effective translation of health research findings into action has been well recognized, but there is evidence to suggest that the practice of knowledge translation (KT) among health researchers is still evolving. Compared to research user stakeholders, researchers (knowledge producers) have been under-studied in this context. The goals of this study were to understand the experiences of health researchers in practicing KT in Manitoba, Canada, and identify their support needs to sustain and increase their participation in KT.

**Methods:**

Qualitative semi-structured interviews were conducted with 26 researchers studying in biomedical; clinical; health systems and services; and social, cultural, environmental and population health research. Interview questions were open-ended and probed participants’ understanding of KT, their experiences in practicing KT, barriers and facilitators to practicing KT, and their needs for KT practice support.

**Results:**

KT was broadly conceptualized across participants. Participants described a range of KT practice experiences, most of which related to dissemination. Participants also expressed a number of negative emotions associated with the practice of KT. Many individual, logistical, and systemic or organizational barriers to practicing KT were identified, which included a lack of institutional support for KT in both academic and non-academic systems. Participants described the presence of good relationships with stakeholders as a critical facilitator for practicing KT. The most commonly identified needs for supporting KT practice were access to education and training, and access to resources to increase awareness and promotion of KT. While there were few major variations in response trends across most areas of health research, the responses of biomedical researchers suggested a unique KT context, reflected by distinct conceptualizations of KT (such as commercialization as a core component), experiences (including frustration and lack of support), and barriers to practicing KT (for example, intellectual property concerns).

**Conclusions:**

The major findings of this study were the continued variations in conceptualization of KT, and persisting support needs that span basic individual to comprehensive systemic change. Expanding the study to additional regions of Canada will present opportunities to compare and contrast the state of KT practice and its influencing factors.

**Electronic supplementary material:**

The online version of this article (10.1186/s12913-017-2573-9) contains supplementary material, which is available to authorized users.

## Background

The transfer of health research findings into practice has traditionally been slow and inefficient, taking up to 17 years for established evidence to reach patients [1, 2]. The result is substandard quality of care, health system inefficiencies, and ultimately reduced length and quality of life [3]. In recognition of this fundamental gap, dedicated efforts to increase use of research evidence to improve health have emerged [4]. No unified term for these efforts exist [5], and appropriate nomenclature continues to evolve, be debated in the literature, and vary by geography [6]. In Canada, “Knowledge Translation” (KT) is the term conventionally assigned to efforts aimed at moving health research into action. Its definition - the synthesis, dissemination, exchange, and ethically-sound application of knowledge to improve health, health service delivery and the healthcare system [7] - is increasingly used worldwide [8, 9]. This definition recognizes that KT is complex and can span the entire research process from conceptualization to implementation. KT is both a science and a practice, with an emerging body of evidence and theory.

The overarching goals of KT have been widely embraced in health research, exemplified by many funding agencies now requiring explicit consideration of KT in their submissions [10]. Such requirements charge health researchers with leading KT efforts, but there is evidence suggesting that KT practice among researchers is progressing in parallel with the discipline. For example, in 2001, a survey of health researchers in Alberta, Canada found that only 15% engaged in interactive forms of research transfer, while the majority relied on traditional dissemination strategies [11]. More recently, in 2014, another survey reported that over 80% of research producers in British Columbia, Canada wanted to learn more about a number of KT skills [12]. Despite more than a decade separating the two surveys, researchers continue to express interest in learning more about basic KT practices such as developing and implementing dissemination plans, as well as more advanced skills like working with decision-makers and developing evidence-informed programs [12].

There is a need for active, theory- and evidence-informed strategies to engage health researchers in practicing KT. Despite their role as key stakeholders in the knowledge-to-action process, researchers have been relatively under-studied in this context. Prevailing models of KT emphasize the importance of preliminary work to explore current practice, gaps, factors influencing behaviour, and adaptation of potential solutions for specific contexts, prior to developing interventions [13].

## Methods

### Study setting and aims

This study was conducted in the Canadian province of Manitoba. Manitoba is the fifth-largest province in Canada, with more than half of approximately 1.2 million residents concentrated in one urban centre. There are four public universities that are all engaged in health research to some degree, with one institution administering doctoral and medical degrees. The goals of this study were to understand the experiences of health researchers in practicing KT in Manitoba, and identify their support needs to sustain and increase their participation in KT. The specific objectives were to explore: (i) how health researchers conceptualize knowledge translation; (ii) KT strategies and activities practiced by health researchers; (iii) barriers and facilitators health researchers have experienced in practicing KT; and (iv) desired supports for facilitating KT practice.

### Conceptual framework and study design

The overarching plan for the study was guided by the Knowledge-to-Action Framework [13], a meta-framework of KT that combines critical features of over 30 planned-action theories and has been adopted by health research agencies worldwide [8, 9]. It is also a process model that provides step-by-step direction for implementation [14]. This study explored the fundamental steps of *identify the problem* (objectives i and ii)*, assess barriers and facilitators to knowledge use* (objective iii)*,* and *tailor to local context* (objective iv) using qualitative descriptive design, a methodology that comprehensively summarizes an issue or event using everyday terms [15]. These steps are considered essential prior to implementing, evaluating, and sustaining knowledge use (in this case, the practice of KT). The qualitative descriptive approach has been previously used in knowledge translation research [16] and is useful for producing practical answers to real-world questions [15].

### Participants

Manitoba health researchers were eligible to participate. A health researcher was defined as someone who spends at least 10% of their working time conducting independent research [11]. A purposive sampling strategy was used, targeting health researchers from the four public universities and those working in academic health sciences organizations via email invitations distributed through institutional listservs. Participants were included from each of the four health research pillars identified by Canada’s national health research funding body: biomedical; clinical; health systems and services; and social, cultural, environmental and population health [17]. Enrollment occurred until data saturation was achieved, broadly informed by previous research suggesting that five to eight participants per health research pillar is likely sufficient to achieve data saturation [18]. A maximum variation approach was used to optimize diversity in career stage and academic discipline to yield a range of perspectives. Following significant interest in the study from health sciences graduate students, six additional trainee participants were sought.

### Data collection and analysis

Individual phone interviews were conducted over a six-week period in 2015 by one member of the research team (CPB). Two pilot interviews were conducted for feedback, one of which was used in data analysis as requested by the participant. Interview questions (Additional file 1) were open-ended and included questions to determine participants’ understanding of KT, their experiences in practicing KT, barriers and facilitators to practicing KT, and needs for KT practice support. Probes were used throughout the interviews where appropriate to explore specific issues in detail and/ or ensure clarity. Interviews were digitally recorded and transcribed verbatim using a commercial service.

The interviewer reviewed transcripts prior to data analysis. Using the objectives as broad categories, four members of the research team (KMS, PLR, BT, KDMW) read, re-read, and open-coded four transcripts, then met to reach consensus on coding schema [19]. Two members of the research team (KMS and PLR) then read and open-coded five additional transcripts and established inter-coder agreement. PLR coded the remaining transcripts, which were collated into themes using NVivo 10 software. Throughout data analysis, codes and themes were discussed and refined with all team members until agreement was reached [20]. Credibility was established through data triangulation and an audit trail. Analyst triangulation was employed, involving multiple analysts discussing and generating key themes [21]. The audit trail was maintained by documenting discussions and decisions made throughout data collection and analysis. Trustworthiness of the data was enhanced through extensive participant quotes.

## Results

### Participants

Twenty-six people participated, following expressions of interest by 49 individuals. The 23 people who were not interviewed were either ineligible (not independent researchers) or did not respond to follow-up e-mails. A description of participants is provided in Table 1. Between five and seven researchers from each health research pillar participated, distributed across trainees, early career (0–5 years), mid-career (6–15 years), and established (more than 15 years) investigators. Most participants were concentrated at one university with the largest health sciences faculty in the province, with four smaller institutions represented by individual participants. This institutional distribution was reflective of the nature of health research in Manitoba. Percentage of work time allocated to research ranged between 10 and 100%, and research areas crossed human and animal populations, disciplines, and health issues. A few participants identified as trainees in addition to being early or mid-career clinical faculty. For the purpose of this study, their responses were considered within the trainee category.

**Table 1:** Participant characteristics (*n* = 26)

Characteristic	Number of participants
Primary Health Research Pillar	
Clinical	8
Biomedical	7
Social, cultural, environmental and population health	6
Health systems and services	5
Career Stage	
Trainee	5
Early (0–5 years)	6
Mid-career (5–14 years)	6
Established (15+ years)	7
Not specified	2
Percent of time spent on research	
25% or less	4
25% - 75%	6
75% or more	14
Not specified	2
Research methodology	
Mostly or all quantitative methods	14
Mostly or all qualitative methods	4
Mixed methods	5
Not specified	3

### Summary of findings

Overall, there were few variations in response trends across health research pillar and career stage. The exception to this were distinct perceptions and KT practice experiences of biomedical researchers, an emergent theme. These distinct views, along with minor variations in perception by career stage, are highlighted as appropriate. Distinct perceptions and opinions about KT also emerged throughout the interviews, as reported in the results.

### Conceptualizing knowledge translation

Overall, KT was broadly conceptualized across participants. Some discussed KT as including multiple components or concepts, while some described a very focused view of KT as a singular construct. No individual expressed a description of KT that included all of the concepts outlined by the group as a whole. Many participants also addressed issues of the value and complexity of KT inherent in their description.

Participants described KT using a variety of synonyms, including implementation, mobilization, and transfer. Across all four research pillars, the most frequently discussed concepts (Table 2) fell into three categories and included: application of research in changing or improving healthcare; dissemination of results at the end of the research process; and working alongside stakeholders to determine research needs, questions, study design, and interpretation of findings. The majority of biomedical researchers included commercialization, industrialization, and/or patents in their conceptualization of KT. Most participants’ descriptions were based on their own experiences or reflection, with a few noting they had no formal KT training or theory. Some noted specific literature or education and the Canadian Institutes of Health Research as references for their definition of KT.

**Table 2:** Common conceptualizations of knowledge translation

KT concept	Representative quote	Pillar & participant
Application of research in changing or improving healthcare	*“…practical use of research findings in the real world”*	Clinical (P16)^a^
	*“…using what we’ve learned from research, to employ it so it gets done in health and social programs and services”*	Social, cultural and environmental health (P05)
Dissemination of results at the end of the research process	*“I’ve been taught… with the idea of knowledge translation, in the way of dissemination, at the end of your grant, when it’s finished”*	Health systems and services (P08)
	*“Knowledge translation is providing the results of scientific studies to end users, and policy makers, and research participants”*	Biomedical (P17)
Working alongside stakeholders at all stages of the research	*“The idea of knowledge translation is just about working together with the people whom you think should be the users of your research, but getting them on the team and on board well before the project even starts to that you can together decide, what should we study, and how should we study it?”*	Social, cultural and environmental health (P11)

^a^P: Refers to individual participants

Half of the participants described KT as either challenging, time-consuming, and/or complex, often recognizing the need for specific expertise. P1 (clinical) said “*I know that, like, sort of all researchers these days are kind of expected to do it, but I don’t think it’s easy to do and everyone can just do it. I think it’s a learned skill [KT], and certainly it’s important to have experts with that skill.”* KT was a relatively new concept for most participants, some of whom discussed how it was a reflection of a changing and evolving culture. P10 (health systems and services) remarked, *“People now value this sort of work [KT], and are calling for it, and are paying for it”*. Others, some with experience in participatory action research and/or evidence-based practice, felt it was simply a new term for existing practices. As P14 (clinical) reflected, *“I recognize that we were already doing… that [KT]. But hadn’t really called it by that name before”.*

The most common discussion of the importance of KT related to the context of relevant and useful research. P20 (social, cultural, environmental and population health) explained that “*Knowledge isn’t really very useful unless it gets into the hands of people who… would apply that knowledge.”* Five participants highlighted the importance of KT by making reference to researchers ‘working in silos’ and the ‘ivory tower’ of academia. As P16 (clinical) put it “*I work in real life settings, where research needs to matter on the ground on a daily basis, not just in the ivory tower of academia”.* More than half of participants considered the practice of KT a professional duty: *“It’s a fundamental requirement of the way that we do business here”* (P11, social, cultural, environmental and population health). Others qualified the value of practicing KT in relation to the specific research context: “*All circumstances and situations are different from researcher to researcher, and the field”* (P4, biomedical).

### Knowledge translation practice experience

As a group, participants described a range of experiences in practicing KT. Almost all participants described using dissemination strategies. Targets of these activities included both traditional academic audiences (through posters, conferences, lectures, and reviews), and non-academic audiences (through videos, social media, websites, and op-eds). Some participants specifically identified a lack of KT practice experience, such as patient engagement, outside of traditional dissemination.

Among those who reported working with stakeholders throughout the research process, advisory groups, relationship-building, and collaborative discussions were employed. A few participants discussed use of participatory action research, including a participant-led photo-voice project. One participant who also held a clinical leadership position described enacting policies in that role to “force” evidence-based medical practice. Some participants discussed conducting retrospective chart reviews as a KT activity, while others described membership in KT organizations or application to KT funding opportunities as indicative of practicing KT. Two participants described participant engagement in a research project, acknowledging the experience as ‘tokenistic’.

In describing both experiences and conceptualizations of KT, some participants associated emotions and emotive states with its practice. These ranged from (in one case) excitement, to fear and intimidation, frustration, burnout (among established researchers) and disappointment. As a group, biomedical researchers expressed concerns about being unsupported in practicing KT, and frustration that the emerging field of knowledge translation is developing with seemingly little regard for discovery research. P2 (biomedical) noted, “*I think I fall into the gap of the scientist that is often forgotten when people talk about KT. In my opinion KT is very biased”,* and “*I feel at times as a basic scientist, I’m an oddball for even trying to do KT, and that it’s very much me morphing what I do to fit preconceived desire(s) for KT”.* To this end, a few felt that the emerging emphasis on KT-related funding opportunities excluded basic research. *“I do have the feeling that there is still lots of basic science and clinical to be done, that doesn’t require this kind of approach, and I worry that, you know, one of the down sides of the emphasis the granting agencies have been putting on knowledge translation – in particular integrated knowledge translation - is that it makes everybody feel like every project has to take this kind of approach”* (P11, biomedical)*.*

### Barriers and facilitators to knowledge translation

Participants identified a myriad of barriers and facilitators to practicing KT. Barriers were predominate, and were classified into systemic or organizational, individual, and logistical barriers. Participants from the biomedical sciences described barriers specific to their field (described below). A small proportion of participants felt there were no barriers to practicing KT.

#### Systemic/ organizational barriers

Among systemic barriers, the most frequently reported barrier related to resource allocation and insufficient funds for practicing KT. Multiple participants felt that that there was a lack of support for practicing KT at the institutional level, be it the University, funder, or government, and that the established nature of academic institutions was a barrier to KT. P3 (biomedical) stated, *“I never found that I got a heck of a lot of any kind of support within the faculty of [X], or within the faculty of [Y] to do that work”.* Time constraints and competing priorities such as tenure, funding, research, publications, and teaching were frequently noted. P16 (clinical) said, *“Sometimes it becomes difficult for you to now reach out into the community, for example, to be able to do that, because of time and resources, and you’re actually… involved in teaching, or in governance at [the] university, or research, and you don’t have that as a focus”*. Several early career researchers felt that being at an early stage was a detriment to KT, due to academic priorities taking precedence and lack of experience. P2 (biomedical) explained, “*As a young investigator, your sole focus in life is getting grants”.* Some participants felt that, in general, KT was not recognized as a priority by academic institutions and governing organizations that support Canadian research programs, or society in general.

#### Individual barriers

Individual barriers to practicing KT were numerous. One of the most commonly identified barriers related to dissemination of research results and concerns over the potential for miscommunication between researchers and stakeholders when engaging with the media*.* This point was summarized by P1 (clinical): *“It’s easy for messages to get skewed a bit in the media, and for people to get the wrong impression”.* Another major barrier identified by participants was their lack of skill or ability in practicing KT. P3 (biomedical) noted: *“I am not ready to do it because I don’t think I have the skill set to do it, not that I don’t think it’s important - just to be clear. I personally don’t have the skills to do it effectively, so I am reticent to do it and waste everybody’s time.”* A few participants expressed disinterest in practicing KT or did not view it as aligning with their research.

#### Logistical barriers

Logistical barriers included difficulties translating a very large body of evidence (and conflicting information), physical barriers when working with rural or remote groups, the current emphasis on training academics to conduct (but not translate) research, and concerns about ethical considerations. For example, some participants expressed concern about Facebook or sharing data when engaging with communities. Two participants from the social, cultural, environmental and population health research pillar mentioned barriers specific to research conducted in rural settings. Namely, they identified weather (flooding), distance (in terms of traveling and the ability to follow up with remote communities), and illness – either theirs personally or public health outbreaks – as barriers to engaging with stakeholders in KT activities in remote communities.

Biomedical researchers noted some barriers specific to the nature of discovery research, namely challenges related to intellectual property concerns and commercialization. P2 succinctly explained, *“If I identify a new drug or a new way to use a drug, and then I want to go to a big pharma company to back the research to move it to the stage where you are using it in a clinical trial, that actually can’t happen. You get no interest or attraction, unless you’ve already protected that property, with a patent or a license”*. P4 described tensions between the desire to share results and the need to obtain a patent: *“the challenge here is that I’m a new investigator, and I need to basically show my research to other people to get more funding and to get more recognition. And… but if I do that, I cannot patent the method anymore.”*

#### Facilitators

Maintaining good relationships with stakeholders was identified as a major facilitator for practicing KT, reflected by trust, honesty, frankness, face-to-face interactions, and understanding each other’s roles. As P11 (social, cultural, environmental and population health) explained; *“the whole thing is based on the relationship with these people, whom we think we want to use our research. And so, that all comes down to relationships, which are built on trust.”* In contrast to comments of participants who reported lack of funds as a barrier to practicing KT, the ability to access resources, such as funding and personnel, was a facilitator for others. One participant described success in acquiring competitive funding to create a video for dissemination of research findings. Mentorship, experience, and training in practicing KT were also identified as facilitators. Several participants noted personal traits –including drive, passion, and enthusiasm—as facilitators for practicing KT. Numerous participants also mentioned how having trained communications personnel working on or alongside their team was a facilitator for practicing KT. As P27 (health systems and services) stated *“I do a [newsletter]… about once a month. I don’t think it would have nearly the polish – and hence the appeal – if I did not have a professional work on it”*. Finally, a few researchers recognized the value of specific local, national, and international platforms or groups in promoting KT – for example, lobbying groups such as the National Institutes of Health.

### Health researcher needs for practicing KT

Participants identified a number of needs they felt, if met, would support their engagement in practicing KT (Table 3). The most commonly identified needs were access to education and training, and access to resources to increase awareness, promotion, and discussion of KT. Participants identified specific preferences for training, including workshops, information sessions, webinars, and mentors. Several researchers were looking for opportunities to collaborate through facilitated networks. Many also noted the need for institutional or funder KT-specific funding policies, small grants, and fellowships, along with continued development of the science of KT. Participants expressed a desire for institutional culture shifts, including recognition for KT efforts in promotion and tenure consideration. Calls for more protected time to practice KT reflected needs for its prioritization.

**Table 3:** KT support needs identified by participants

Type of need	Example
Resources	
Funding	KT-specific grants, subsidies
Expertise/personnel	KT consultants or team members, communications specialists, clinical research associates, statisticians
Time	Protected time (academic appointments)
Support	
Recognition	Promotion and tenure
Education and training	Workshops, info sessions, webinars, ‘KT 101’, graduate courses, skills assessments for team members
Leadership and mentoring	Institutional leaders, aggressive and active involvement in KT
Institutional/cultural changes	Improved KT infrastructure (e.g.: access to information), structural changes (e.g.: pay-for-performance healthcare model)
Opportunities	
Collaboration and networking	Facilitated networks, provider engagement, large consortiums
Experience in KT	Hands-on KT, engaging in process, experiencing KT successes
Research	
Advance the science of KT	Finding gaps, context-specific KT strategies, evaluation and maintenance of KT strategies, systems research
Promotion of KT	
Increased awareness, communication, discussion of KT	Regular events, social media development opportunities, understanding roles and expectations between researchers and stakeholders

## Discussion

Health researchers represent a critical and understudied KT stakeholder group. This small study makes an important contribution to knowledge translation science by identifying current issues in KT practice among health researchers in Manitoba, Canada, highlighting opportunities to advance the intersection of KT practice and science. The findings also highlight important distinctions between health research pillars with implications for KT scientists and practitioners. Collectively, the results of this study point to a number of next steps for advancing KT practice in health research.

A key finding of the study was the broad range - yet distinct and personalized - conceptualizations of KT across researchers. This finding was consistent with previous studies who described strong variations in individual understandings of KT [22]. Such varied conceptualizations of what constitutes KT are not surprising given the lack of a consistent, agreed-upon conceptualization of KT within the scholarly field itself [5, 13]. For example, some authors and definitions distinguish commercialization from knowledge translation [7, 23], while others consider translation from discovery to diagnostic and intervention applications to practices and policy along the same continuum [24]. These distinct views serve as a challenge to KT scholars to ratify a comprehensive definition capturing the full scope of knowledge translation in health research.

With regards to reported KT practices, in this study most participants acknowledged that there was more to KT than they had included in their own research programs. Although it is not possible to determine in this study what appropriate KT practice is for each individual and their specific project(s), this finding is noteworthy because it suggests that there may be potential to enhance or expand the practice of KT among health researchers. Furthermore, the emergence of negative emotions associated with practicing KT among some participants is also a noteworthy consideration for KT practitioners. Reported feelings of frustration and burnout were from established researchers, whereas those expressing excitement and fear were in the early career stage. Though not specifically identified as a barrier by participants, negative emotions associated with KT may reduce researchers’ involvement in practicing KT, due to the association between emotions and decision-making processes [25].

Finally, the absence of discussion about certain KT practice issues is important. The majority of participants did not make any mention of evaluation of KT practice, indicating that this recognized component of the knowledge-to-action process was not included in their current conceptualization of KT. It might suggest that researchers view the practice of KT evaluation as outside of “research”. Others have also reported a lack of awareness of evaluation strategies for KT activities among researchers [26], indicating this is not an isolated finding. This is an important gap given the need to advance KT methodologies and interventions, reflecting a missed opportunity that more researchers could be using to study and share the effectiveness of their KT efforts and leverage rigorous approaches into peer-reviewed publications.

The examination of barriers and facilitators to practicing KT is critical for tailoring any future interventions to specifically address these factors [4]. Many of the barriers identified in this study have been previously expressed by research participants (both researchers and end-users) in studies throughout Canada [11, 12] and globally [22, 27–29]. The persistence of reported barriers over time is important, and suggests there is still opportunity to improve. The systemic and organizational barriers that persist require ongoing coordinated efforts by academic institutions, research funders, and health policy-makers. Continued work is needed to address these critical issues. A key barrier noted in this study was the lack of academic recognition for practicing KT. While compelling, this issue was recognized as early as 1999 in a Canadian Health Services Research Foundation workshop report that explored issues in linkage and exchange between researchers and decision-makers [30]. Though changes in academic recognition of KT practices are emerging [31, 32], this barrier continues to persist around the world [28]. Where recognition does exist, the extent to which these policies are promoted, recognized, applied, and evaluated are unclear. Many of the identified support needs for practicing KT echo those of similar studies [12, 22, 26, 33] and highlight that there continues to be a need for foundational capacity building for novice KT practitioners, as well as the larger institutional and organizational supports for researchers who are highly invested in practicing KT. Some noted support needs, such as training and mentorship opportunities, are already undergoing active development throughout Canada and around the world in efforts to actively address this issue [12, 34–41]. Others, such as intellectual property barriers described by biomedical researchers, pose unique challenges for those working to promote and advance KT science and practice.

The distinct barriers described by biomedical researchers contributed to a unique KT context experienced by this group relative to other health researchers in this study. Biomedical researchers also described discrete conceptualizations of KT and perceptions of its practice. This unique biomedical KT context is a critical finding because biomedical research makes up the majority of health research applications received and funded by Canada’s federal funding body [42]. The biomedical researchers in this study did not question the value of KT, which reflects a shift from a previous survey of KT activities and perceptions of Alberta health researchers in which applied scientists reported higher perceived value of KT activities then basic scientists [11]. Rather, the overall interpretation of KT and view of its conduct were clearly different from the other three pillars. This differential view may have contributed to feelings of being “left out” or “forgotten” in the world of KT described by some participants. Existing scholarship on KT in the biomedical context is largely limited to factors influencing the junction between fundamental discoveries and clinical medicine only [29], and can omit implementation considerations that may be indirectly impacted by basic science. As noted above, while published calls for policy and systemic changes to facilitate KT exist, there is little evidence of action or evaluation of impact. There remains much opportunity to advance the practice of KT in basic science, and in particular to bring researchers and practitioners together across all components of the translational continuum to move out of the so-called research “death valleys” that persist and divide traditional domains of health research and practice [43].

### Limitations

Categorization of research focus using the Canadian health funder’s “pillar” system is an arbitrary designation, and many participants identified with more than one pillar but were required to select only one. Though this research examines a broad group of researchers at various career stages and sectors of Canadian health research, limitations exist in terms of the relatively small sample size and sampling strategy – targeting individuals who are already aware of, interested in, and potentially practicing of KT. It is reflective of only one Canadian region, and is not a national sample. Most respondents were from a single institution, although this reflects the distribution of health research conducted in Manitoba. However, the results suggest that the evolution of KT practice is ongoing and not uniform throughout Canada, highlighting the need for an expanded and comparative analysis of the state of KT practice in Canadian health research.

## Conclusions

Published calls for KT in health research [4, 44] reflect passive diffusion and are unlikely to affect measurable behaviour change among health researchers [45]. Skepticism about the value of KT [46] has been postulated to reflect a lack of cohesion in the relationships between evidence producers (researchers) and users (clinicians, policy-makers, and the public) [47]. The major findings of this study were the continued variations in conceptualization of knowledge translation across individual researchers and pillars of health research, and persisting support needs that span basic individual to comprehensive systemic change in one Canadian province. In addition, reaching consensus on a clear and comprehensive definition of KT will have a significant impact on advancing the science and practice of the field. Expanding the study to additional regions of Canada will present opportunities to compare and contrast the current state of KT practice and its influencing factors.

## Additional file

Additional file 1: Interview questions – open-ended qualitative interview questions. (DOCX 17 kb)

## Abbreviations

KT: Knowledge translation
