# Supplementary material for: A descriptive qualitative examination of knowledge translation practice among health researchers in Manitoba, Canada
Source: BMC Health Serv Res. 2017 Sep 6;17:627. doi: 10.1186/s12913-017-2573-9 (PMC5585925; doi:10.1186/s12913-017-2573-9)
Supplement: Additional file 1: — Interview questions – open-ended qualitative interview questions. (DOCX 17 kb) [file 12913_2017_2573_MOESM1_ESM.docx]

1. To begin, can you tell me a little bit about your research background and your work?
   1. Sub questions regarding career:
      1. What is your primary academic appointment? (assistant professor, associate professor, professor, community/healthcare researcher with no academic appointment, other)
      2. What is your home institution, faculty, and department?
      3. What is your level of experience and/or career stage?
      4. How many years have you worked as an independent investigator?
      5. What percent of your time do you spend on research?
   2. Sub questions regarding research:
      1. What is your research population?
      2. What is your research setting?
      3. What is your primary methodology (i.e. qualitative/quantitative/both)?
2. As you know, we are interested in knowledge translation, also known as knowledge exchange, knowledge transfer, implementation science. Before we go further, I’m interested to hear how you define or think about the concept of “knowledge translation”?
   1. Sub questions:
      1. How important is knowledge translation to your work?
3. What is your definition of knowledge translation based on?
   1. Sub questions:
      1. Are there any underlying principles or theories that lead to that definition? Can you describe them?
      2. Where and how did you learn about knowledge translation?
4. What experiences have you had in conducting or “doing” knowledge translation?
   1. Sub questions:
      1. How successful or unsuccessful were those experiences?
      2. What do you see as the barriers (and/ or facilitators) to your success (or lack of success) in those experiences?
5. Broadly speaking, what factors do you see impacting your desire and/or ability to engage in knowledge translation in your future work?
6. What changes/ developments/ resources could be made that would help you better use (or engage) in knowledge translation in your work?
   1. Sub questions:
      1. Are there any specific issues or components of knowledge translation you would like to integrate but are not?
      2. What support strategies would be of most benefit to you?
7. Is there anything any else related to your involvement in knowledge translation (or knowledge translation in general) that you think is important for me to know?
